# Mechanical Properties Analysis of Nickel-Based Composite Coatings Prepared by Laser Cladding

**DOI:** 10.3390/ma18235381

**Published:** 2025-11-28

**Authors:** Shaoping Hu, Longfeng Sun, Yanchong Gao, Chao Zhang, Tianbiao Yu

**Affiliations:** 1 School of Mechanical Engineering and Automation, Northeastern University, Shenyang 110819, China; 2190007@stu.neu.edu.cn (S.H.); 18340355586@163.com (Y.G.); zhangchao1@mail.neu.edu.cn (C.Z.); 2 Jiangnan Shipyard (Group) Co., Ltd., Shanghai 201913, China; 18945068082@163.com

**Keywords:** laser cladding, mechanical properties, IN718 alloy, EA4T steel

## Abstract

During the laser cladding process for composite coatings, significant differences exist in the physical and mechanical properties between the substrate and the composite coating materials. Therefore, a systematic analysis of the mechanical properties is necessary to mitigate issues such as cracking and deformation caused by performance mismatch. This study investigated the mechanical properties (microhardness, wear resistance, tensile strength) of composite coatings formed by laser cladding IN718 alloy onto an EA4T steel substrate. Given the critical influence of scanning strategies on cladding layer quality, this study also examined the relationship between the tensile direction and scanning direction. By analyzing mechanical responses under different orientations, it revealed the patterns of influence on tensile properties and anisotropy characteristics of the cladding layer, providing a theoretical basis and process guidance for achieving high-performance cladding layers. Tensile tests conducted at different angles on the IN718 cladding layer indicate that when a thin cladding layer is required, selecting a scanning speed direction parallel to the primary tensile direction yields superior results. Conversely, for applications demanding a thicker cladding layer, aligning the scanning direction perpendicular to the tensile direction better leverages the cladding layer’s performance.

## 1. Introduction

Laser cladding, as a green remanufacturing surface engineering technology, operates on the principle of using a high-energy laser beam as a heat source to deposit metal alloy powder (or wire) onto the substrate surface through synchronous or pre-set methods. Under the thermal effects of the laser, the substrate surface layer and coating material undergo rapid melting to form a metallurgical melt pool. Following directional solidification, this process yields a dense functional coating with metallurgical bonding to the substrate and controllable thickness [1].

In mechanical equipment, friction and wear are major causes of component failure and significantly impact equipment precision. Laser cladding technology enables the repair and reuse of parts that have been worn to the point of scrapping. Additionally, the wear resistance of the clad layer can be intentionally adjusted by selecting the cladding material, ultimately achieving greater wear resistance than the substrate [2,3]. Zhu et al. [4] selected 70% Ni60A + 30% WC as the cladding material. Their research revealed that WC not only enhanced the solid solubility of alloying elements in the cladding layer but also increased its microhardness. The wear mechanism of the cladding layer shifted from predominantly adhesive wear to primarily abrasive wear. Yang et al. [5] investigated the wear resistance of various WC/NiCrBSi metal matrix composite coatings. The mass loss increased and the average friction coefficient decreased with increasing load from 60 to 260 N for the NiCrBSi + 60% agglomerate WC composite coating. Accordingly, the wear mechanism changes from adhesion to abrasion. Zhang Yu et al. [6] employed ultra-high-speed laser processing to produce nickel-based WC coatings with denser microstructures, finding that ultra-high-speed lasers yielded superior surface quality compared to low-speed lasers while minimizing heat effect on the substrate. Kong et al. [7] observed that ultrafine WC particles significantly refined the grain structure, resulting in a friction coefficient and mass loss of 4/5 and 3/4 than that of the substrate, respectively. Furthermore, the hardness of the clad layer exceeded the substrate by 50 HV_0.2_ after 6 h of tempering at 600 °C. Chen et al. [8] prepared Co-WC composite coatings on IN718 alloy surfaces via laser cladding. They observed coating hardness proportional to WC content and significantly reduced wear rates compared to the substrate. The wear mechanism of the composite coatings at 600 °C changed from abrasive and adhesive wear to oxidation wear, and the COF and wear rate were reduced by 32.4% and 57.3%, respectively. Li et al. [9] analyzed the microstructure and properties of nickel-based powders with varying WC contents. Results indicated that increasing WC content led to finer grains and higher average hardness in the cladding layer. The friction coefficient exhibited an inverse trend to average hardness, gradually decreasing with increasing WC content. Wei et al. [10] observed that WC addition retarded columnar grain growth, with hardness positively correlated to WC content. The wear mechanism of NiCrAl-WC coatings is the combination of adhesive wear, abrasive wear, and oxidation wear, in which the NiCrAl-40%WC coating has the best wear resistance due to its high WC content. Kyekyere et al. [11] investigated the influence of TiC and SiC on the wear behavior of laser-clad 16MnCr5 steel-based composites. Compared to the 16MnCr5 alloy, the TiC-16MnCr5 composite exhibited a 1.69 times reduction in volumetric wear loss, while the SiC-16MnCr5 composite demonstrated a 1.28 time decrease in volumetric wear loss. Aprameya et al. [12] developed SS316 and Mo-enhanced composite cladding layers via direct laser energy deposition on SS304 substrates to enhance high-temperature wear resistance. Compared to the SS316 cladding, the Mo-containing SS316 cladding exhibited higher hardness, promoting the formation of stable oxide films. This transitioned the wear mechanism from severe adhesive wear to a more stable oxidative wear process. Additionally, Aprameya et al. [13] developed a Mo-enhanced Stellite 6 cladding using laser directed energy deposition technology to provide enhanced surface protection. Wear results indicate that at room temperature, the cladding primarily exhibits abrasive wear accompanied by minor plastic deformation. However, at 600 °C, the wear mechanism evolves into a composite pattern of severe adhesive wear, oxidative wear, abrasive wear, and plastic deformation, with oxidative wear dominating the tribological behavior.

Tensile testing of clad layers investigates their tensile properties, yielding experimental data such as tensile strength, elongation, and reduction of area. Wang Z et al. [14] investigated the influence of process parameters on the mechanical properties of 304L stainless steel coatings produced via laser directed energy deposition. Experimental results indicated that lower linear heat input yielded higher tensile strength, yield strength, and elongation compared to higher linear heat input, while longitudinal ductility was lower than transverse ductility. This was attributed to finer microstructures formed by lower linear heat input, which enhanced the material properties. Chen Z et al. [15] investigated structural and property changes in laser-cladding coatings containing 10%, 30%, and 50% nickel-coated carbon nanotubes by mass fraction. Results revealed that most carbon nanotubes in the cladding layer transformed into carbon nanoproducts such as graphene nanosheets, graphene fragments, carbon nanoribbons, and diamond-like nanoparticles. Wear resistance also improved with Ni-CNT addition, with tensile properties peaking at 30% mass fraction, while wear resistance was optimized at 50%. Zhang et al. [16] examined the microstructure and properties of laser-clad Ti-25V-15Cr-0.2Si coatings on a Ti-6Al-4V substrate. Test results showed that the laser-cladding Ti-25V-15Cr-0.2Si coating exhibited tensile strength exceeding 900 MPa and elongation greater than 9%. Lambarri et al. [17] employed a continuous-wave Nd: YAG laser for cladding on Inconel 718 substrates, identifying columnar crystal structures and Laves phases within the coating. The Laves phase, exhibiting lower toughness compared to columnar crystal structures, negatively impacts the overall coating performance, significantly reducing tensile strength under tensile loading. Wen et al. [18] studied the microstructure, tensile strength, and corrosion resistance by laser cladding a proprietary intensified dual-phase steel (IDP) powder onto a 2205 stainless steel substrate. The presence of Ni elements induced the formation of the Fe_3_Ni_2_ phase in the clad layer, increasing hardness by 15% compared to the substrate. Tensile strength along the scanning direction ranged from 850 to 1000 MPa, while perpendicular to the scanning direction, the tensile strength was 700–800 MPa with an elongation of 40%. Dharavathu et al. [19] experimentally compared the effects of conventional laser melting and high-speed laser cladding on the microstructural evolution and mechanical properties of clad layers. Results revealed that the accelerated cooling rate and microstructural refinement induced by high-speed laser cladding increased the tensile residual stress by 65% and reduced the tensile strength by 25%. Horisawa et al. [20] investigated the mechanical behavior of steel structural members using arc thermal spray cladding layers. The thermal spraying process employed martensitic stainless-steel wire to achieve a target cladding thickness of approximately 5 mm. Tensile tests indicated that the cladding exhibited an estimated tensile strength of 231 MPa, with brittle fracture occurring at 0.3% strain. Despite its limited tensile ductility, the cladding contributed to increased axial stiffness within the elastic range.

This study investigated the mechanical properties of composite coatings formed by laser cladding IN718 alloy onto an EA4T steel substrate. By incorporating varying proportions of WC particles as reinforcing phases, the enhancement mechanisms for properties such as hardness and wear resistance were explored. Simultaneously, the relationship between tensile properties and the scanning angle was investigated, revealing the variation patterns of tensile performance and anisotropy characteristics in the clad layer. This provides theoretical basis and process guidance for achieving high-performance cladding layer fabrication.

While previous studies on laser-clad IN718-WC composites have primarily focused on hardness and wear resistance, the novelty of this work lies in the systematic and concurrent investigation of microhardness, wear performance, and tensile properties—particularly the anisotropy of tensile behavior relative to the cladding scan direction. This integrated approach provides comprehensive mechanical property data and process guidance for fabricating high-performance cladding layers under specific service requirements, which has been less explored in prior literature.

## 2. Materials and Methods

**Figure 1:**
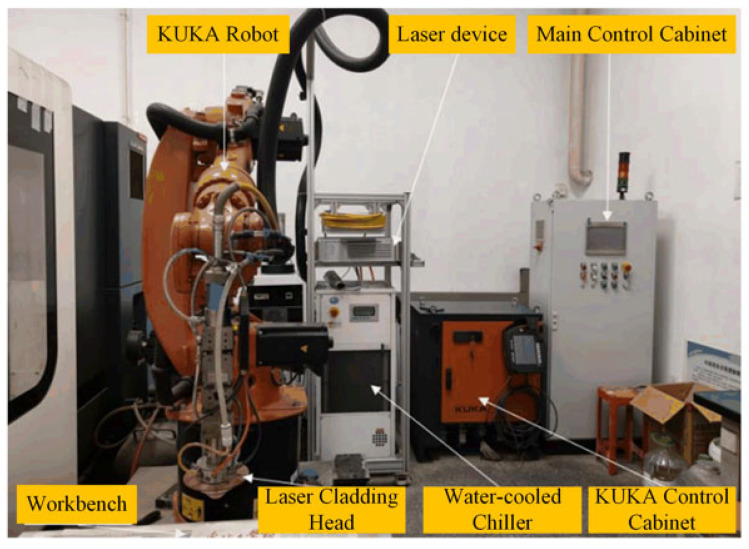
Laser cladding experimental platform [21].

Laser cladding technology involves melting cladding material using a high-energy laser beam to deposit it onto the surface of the substrate, forming a strong metallurgical bond with the base material to achieve coating reinforcement or repair objectives. This technique is widely applied in the repair and manufacturing of critical components. The laser cladding experimental platform is shown in Figure 1 [21].

In this study, EA4T steel and IN718 were selected as the substrate and cladding layer materials, respectively. EA4T steel is a low-alloy structural steel characterized by its combination of high strength and high toughness. Its excellent mechanical properties, high corrosion resistance, and acceptable impact strength make it particularly suitable for components subjected to high dynamic loads during service. The chemical composition of EA4T is shown in Table 1 [21].

IN718 is a nickel-based high-temperature alloy powder widely used in laser powder bed fusion technology. This powder forms high-quality metallurgical bonds with various substrates, featuring low dilution rates and dense cladding that effectively minimizes cracks and porosity. IN718 exhibits high fatigue strength and resistance to stress corrosion cracking, making it suitable for vibrating and high-temperature environments. It also possesses excellent machinability, facilitating subsequent precision machining and forming. The chemical composition of IN718 is shown in Table 2 [21].

In studies on the laser cladding of IN718, numerous researchers have investigated the effects of adding various substances—such as common WC, TiC, and rare earth elements—on clad layer properties [4,5]. WC significantly enhances the hardness of the cladding layer, and unmelted WC particles can also provide structural support in friction environments, thereby reducing the coefficient of friction. Therefore, WC was selected as the hardness-enhancing material to strengthen the cladding layer. However, different WC addition levels exhibited varying effects on IN718 hardness, necessitating the establishment of a step-content design to investigate the influence of different WC concentrations on cladding layer hardness. Three composite materials containing 5%, 10%, and 15% WC by mass fraction were prepared and compared with the base material and IN718 material in terms of hardness. This allows for the observation of the effect of different WC contents on the hardness and identification of the content that most closely matches the hardness of the base material.

**Figure 2:**
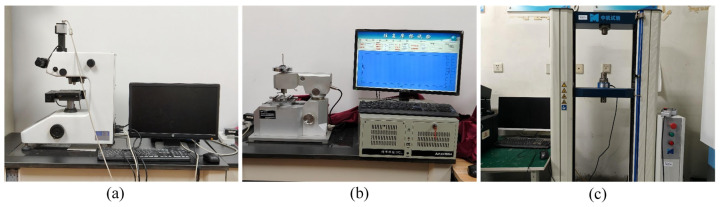
Testing equipment: (**a**) MH-500 hardness tester; (**b**) MFT-4000 multi-functional test machine; (**c**) WDW-100E electronic universal testing machine.

The cladding layer with a WC mass fraction of 10% was selected to investigate the mechanism influencing hardness. This composition exhibited an optimal window in macroscopic performance, making its microstructural investigation most valuable and representative. The in-depth analysis of this optimal composition will provide clearer insights into the strengthening mechanism of WC, offering greater guidance than selecting a composition with mediocre or excessive properties. Hardness measurements were taken both away from the WC particles and close to them. The difference in hardness between these two locations was further analyzed to investigate the hardening mechanism of WC. The equipment used in the experiment was the MH-500 produced by Shanghai Hengyi Precision Instrument Co., Ltd. (Shanghai, China), primarily designed for measuring the surface layers and microhardness of metals and alloys. The measurement accuracy complies with the American Society for Testing and Materials ASTM E-384 standards. Its physical appearance is shown in Figure 2a.

The analysis of friction and wear properties for laser cladding layers is crucial in practical applications, particularly where high surface wear resistance is required. The MFT-4000 (Lanzhou Huahui Instrument Technology Co., Ltd., Lanzhou, China) is a multifunctional experimental device for testing the tribological properties of materials, as shown in Figure 2b. It is widely used to evaluate the friction, wear, and performance of materials under lubricated conditions, commonly employed in laboratories and industrial research to optimize material surface properties. For this experiment, a reciprocating friction test was selected to obtain accurate experimental data and clearly observable post-friction wear morphology. The counterpart material was Al_2_O_3_. The test parameters were set as follows: load of 10 N, speed of 220 mm/min, and friction length of 5 mm. To ensure the reliability and reproducibility of the friction and wear data, each material composition was tested three times under identical conditions, and the average value of the coefficient of friction was reported.

Friction and wear tests were conducted on the substrate material EA4T steel and the cladding layer material IN718 under the aforementioned parameters. The resulting scratches were examined using an Olympus OLS4100 device (Olympus Corporation, Tokyo, Japan) to observe their size and characteristics. Since WC particles were previously added to increase cladding hardness, prioritizing the influence of WC content on friction and wear was chosen to avoid introducing excessive reinforcing phases that could complicate the composition. The microstructure was characterized using SEM, FEI QUANTA 600 (FEI Company, Hillsboro, OR, USA).

Tensile properties serve as a crucial metric for evaluating metal performance, providing essential guidance for determining material suitability across diverse usage scenarios. The experimental equipment was the WDW-100E electronic universal testing machine (Wuxi Fosde Instrument Equipment Co., Ltd., Wuxi, China, Figure 2c), used for evaluating the mechanical properties of materials. It is multifunctional and capable of performing various tests, such as tensile and bending tests. It features high precision, high sensitivity, and excellent stability. Its technical parameters are shown in Table 3.

**Figure 3:**
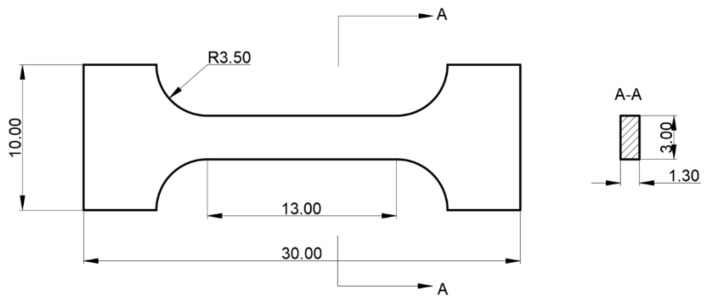
Dimensions of tensioned parts.

To minimize time and material expenditure while obtaining accurate tensile data, smaller-sized standard tensile specimens were selected for subsequent tensile testing. The dimensions of the tensile specimens were designed according to standards, as shown in Figure 3.

**Figure 4:**
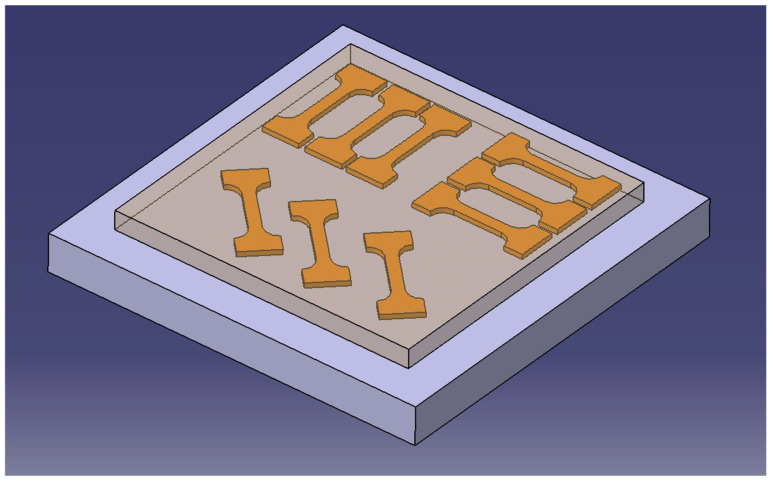
Schematic diagram of cutting of stretched parts.

For this study, experiments were designed by first performing laser cladding using the optimal process parameters obtained previously: P = 460 W, V_s_ = 7 mm/s, V_f_ = 1.1 r/min. To avoid excessive heat accumulation, unidirectional scanning was selected for the cladding process. To investigate mechanical property variations in different directions, the laser-clad layers were cut at 0°, 45°, and 90° relative to the scanning direction, as illustrated in Figure 4. Additionally, to study the tensile properties of the substrate material, three tensile specimens were taken from the substrate section. Since EA4T axle steel is manufactured entirely through forging, the influence of material anisotropy on properties in different directions was not considered.

## 3. Results and Discussion

### 3.1. Microstructural Characterization

**Figure 5:**
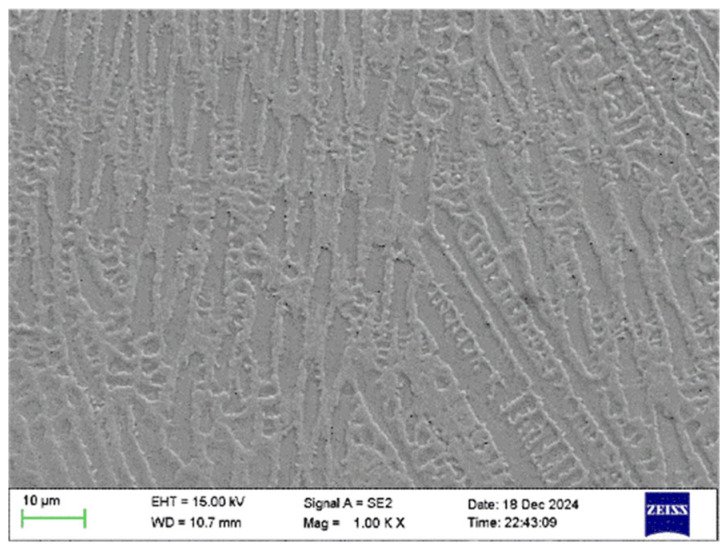
SEM image of melt pool morphology after corrosion.

During dendrite growth, element segregation primarily results from differences in solubility and diffusion rates of various elements during the rapid cooling and solidification of the molten pool. Element segregation significantly impacts microstructural properties, making its study essential. In this section, the microstructural characterization was performed on a reference sample of pure IN718 cladding without any WC addition. This sample was specifically prepared and analyzed to establish a baseline for understanding the intrinsic dendritic solidification behavior, elemental segregation patterns (particularly of Nb), and phase formation (such as the δ-phase and Laves phase) in the IN718 matrix material under our laser cladding parameters. This baseline was deemed essential before introducing the complicating factor of WC reinforcement to cleanly distinguish the effects inherent to IN718 from those induced by WC. As observed in the SEM images (Figure 5), the etched molten pool exhibited an uneven surface. The columnar crystalline structure was severely etched by the solution, forming pits, while the interstitial spaces between the columns demonstrated greater corrosion resistance and thus showed less etching.

**Figure 6:**
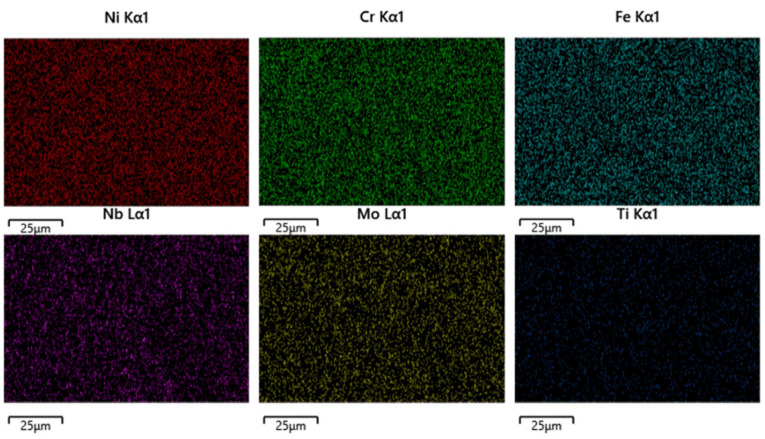
Molten Pool EDS Scan Results.

In IN718 material, element segregation readily forms harmful phases such as the δ phase (Ni_3_Nb). The δ phase exists as a precipitation in the alloy, and severe niobium segregation in the molten pool can lead to excessive δ phase precipitation. The formation of the δ phase significantly compromises the alloy’s high-temperature tensile strength and fatigue properties. In addition to the δ phase, other phases are also formed. To investigate the coexistence of the δ phase with other phases, an EDS scan analysis was performed on the sample shown in Figure 6.

**Figure 7:**
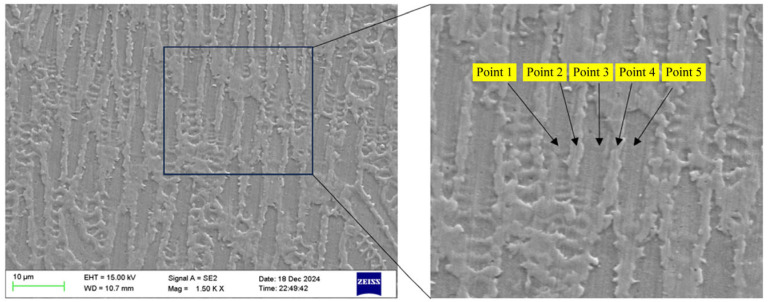
Analyzing elemental composition pickup point locations.

Corrosion-etched scanning results revealed that among the aforementioned elements, Ni, Cr, Fe, Mo, and Ti exhibited minimal segregation while Nb exhibited segregation patterns similar to the dendritic morphology observed via SEM. To further investigate the influence of dendritic growth on the segregation of these elements, points were selected in Figure 5 to analyze their elemental composition. Points 1, 3, and 5 are located within the columnar grain structure, while points 2 and 4 are situated in the precipitation structure within the columnar grain boundaries. The locations of the selected points are shown in Figure 7.

EDS analysis was performed on the five points shown in the figure above to determine the Ni, Cr, Fe, Mo, Ti, and Nb elemental contents in the IN718 material. The final results are presented in Table 4.

As shown in Table 1, many elements exhibited uneven distribution during laser cladding due to dendrite growth. The average Ni content within dendrites was 52.71%, while the average content in the inter-dendritic structure was 50.17%. Cr and Ni exhibited similar trends in content variation between dendrites and inter-dendritic regions. This is because both elements form γ-phase with good solid solubility, resulting in higher dendrite-internal content than inter-dendritic content. Fe content is also significantly higher within dendrites than in the interstitial regions. One reason is Fe’s relatively high solid solubility, allowing it to distribute uniformly in the alloy matrix (γ phase). Additionally, Fe forms a matrix structure with Ni, contributing to its relatively high concentration within dendrites. Mo exhibited high solubility in IN718 material, but its solubility decreased with decreasing temperature. Consequently, Mo segregation during solidification led to its accumulation in dendritic interstices. Mo enhanced the alloy’s high-temperature strength; higher Mo content strengthens grain boundaries and improves alloy stability. Ti exhibited low solubility in the IN718 alloy. As the temperature decreased, its solubility gradually declined, leading to segregation into the dendritic interstices.

Consequently, Ti content was lower within dendrites and higher in the interstices. At elevated temperatures, Ti significantly enhanced alloy strength but simultaneously adversely affected plasticity and toughness. Nb exhibited the most pronounced segregation among elements, with an average mass fraction of 2.73% in dendrites and 11.12% in inter-dendritic voids. Nb’s low solid solubility at low temperatures causes its concentration to decrease as temperatures drop and dendrites grow, ultimately leading to segregation into inter-dendritic voids. Consequently, the intergranular content is significantly higher than the intragranular content. Nb precipitation typically forms the δ-phase and Laves-phase in intergranular regions. These phases are relatively stable at elevated temperatures, which enhances the material’s high-temperature strength and creep resistance. However, this precipitation simultaneously reduces the alloy’s ductility and toughness.

### 3.2. Hardness Test Results

**Figure 8:**
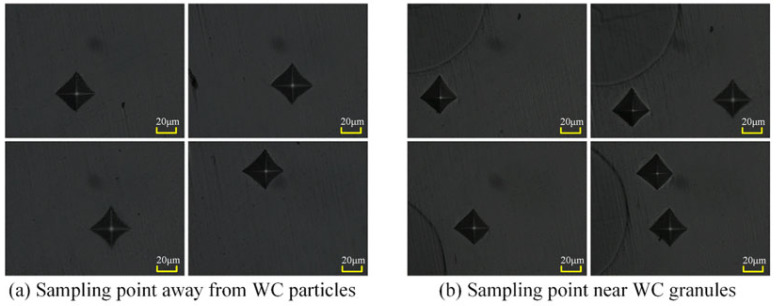
Sampling points for hardness measurements.

Hardness measurements were conducted on each material according to the aforementioned test protocol. To obtain more accurate hardness values, five measurement points were selected for each material requiring hardness data, and the average hardness was calculated. For the hardness of the surrounding structure around WC particles, measurement points should be positioned as close as possible to the WC particles without making contact. The sampling points are illustrated in Figure 8.

**Figure 9:**
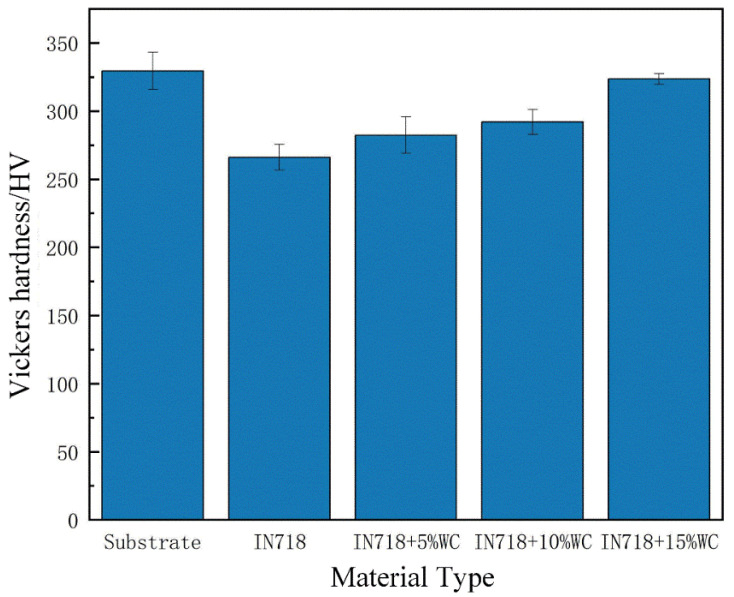
Hardness of substrate and cladding layers with different WC contents.

The final hardness values for each test group are shown in Figure 9. Significant hardness differences existed between the base material EA4T steel and the IN718 material. Measurements indicate that EA4T steel exhibited a hardness of 328.4 HV, while the laser cladding IN718 material achieved 262.8 HV. The hardness gradually increased with the addition of WC, rising from 282.9 HV to 324.8 HV. The maximum hardness was achieved at a WC mass fraction of 15%, which was essentially consistent with the hardness of the base material EA4T steel.

For regions distant from the WC, increased WC content led to higher hardness. This is primarily because smaller WC particles undergo complete melting during laser cladding, decomposing upon mixing with IN718 to form W and C elements. These elements dissolve into the IN718 matrix, forming a solid solution. The solid solution distorts the matrix crystal lattice, thereby enhancing the material’s hardness and strength.

Measurements revealed a significant hardness difference between the microstructure away from WC particles and that near the WC particles at a WC mass fraction of 10%. The average hardness of the microstructure away from WC particles was 285.3 HV, while that near WC particles averaged 325.6 HV, representing a difference of 40.3 HV. Given the flow of the molten pool during laser cladding and the absence of deformation in the observed WC particles, it can be concluded that all microstructural components within the clad layer, except for the unmelted WC particles, are consistent in composition. Therefore, it can be inferred that the significant hardness variation within the same microstructure is attributable to the WC particles.

First, during laser cladding, the material undergoes an instantaneous transition from solid to liquid state before cooling and solidifying. The presence of WC particles during this cooling and solidification process causes the surrounding structure to solidify with a finer grain size compared to other areas. The temperature gradient during the transition from liquid to solid state in the cladding process is the key factor determining the fineness of the clad layer’s microstructure. Consequently, the structure becomes denser, resulting in superior mechanical properties. Second, when measuring the hardness of the matrix surrounding WC particles, the indentation size measurement at a distance causes deformation in the surrounding matrix. The presence of WC particles as a second phase inhibits this deformation, increasing deformation resistance and thereby further enhancing the hardness of the substrate.

### 3.3. Friction and Wear Test Results and Conclusions

**Figure 10:**
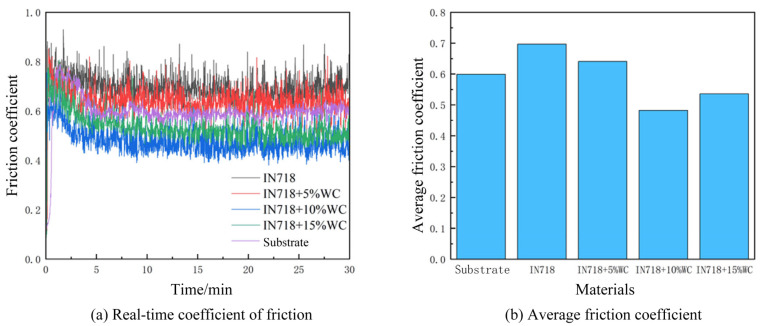
Real-time friction coefficient and average friction coefficient.

The aforementioned materials were tested on a friction and wear testing apparatus. Test process data and results were plotted using Origin software, yielding the variation of the friction coefficient over time and the average friction coefficient during the friction and wear process, as shown in Figure 10.

As shown in Figure 10, the coefficient of friction initially increased over time before decreasing and eventually stabilizing. The base material exhibited a lower friction coefficient compared to IN718. Testing revealed an average friction coefficient of 0.697 for the IN718 cladding layer. This coefficient gradually decreased with increasing WC content, reaching a minimum of 0.482 at a WC mass fraction of 10%. As WC content continued to increase, the average friction coefficient rose to 0.536. At a WC particle content of 5%, the insufficient particle quantity failed to form effective support within the cladding layer. Although the friction coefficient decreased relative to pure IN718 material, the substrate material remained the primary factor contributing to friction and wear. At 15% WC content, the relatively higher particle density impaired fluidity within the molten pool. This reduced fluidity readily led to stress concentration during cladding layer cooling, exacerbating wear. At a WC mass fraction of 10%, the optimal ratio ensured that smaller-radius WC particles fully melted and thoroughly mixed with the IN718 material, enhancing the wear resistance of the cladding layer. Additionally, heat caused the surfaces of larger-radius WC particles to partially dissolve, promoting material bonding. Finally, the thermal expansion coefficient is a critical factor in material bonding. The optimal ratio allows IN718 to compensate for stress concentrations caused by differing thermal expansion coefficients through plastic deformation. Conversely, higher WC content reduces the proportion of the base material, compromising its continuity and increasing susceptibility to stress concentration and micro-cracks, which adversely affect microstructural properties.

**Figure 11:**
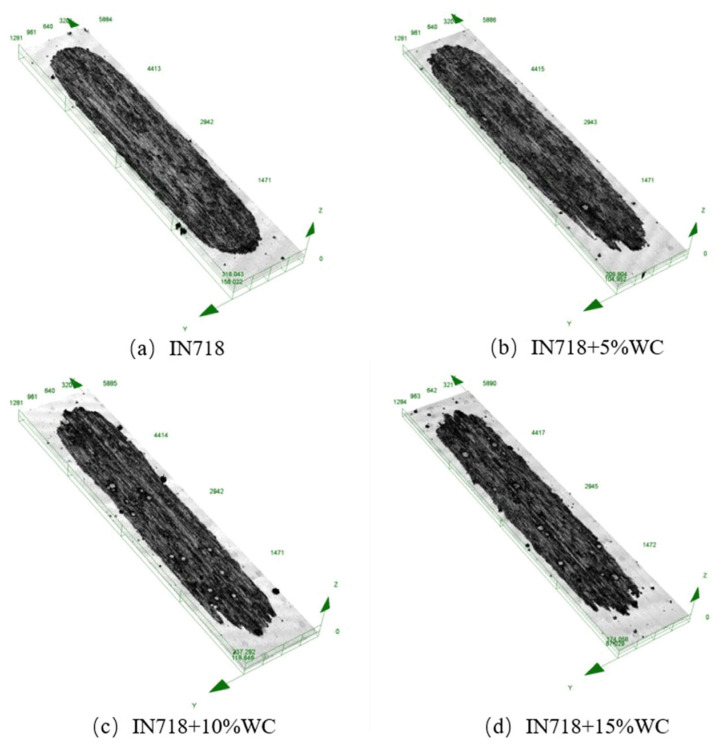
Surface morphology after wear.

**Figure 12:**
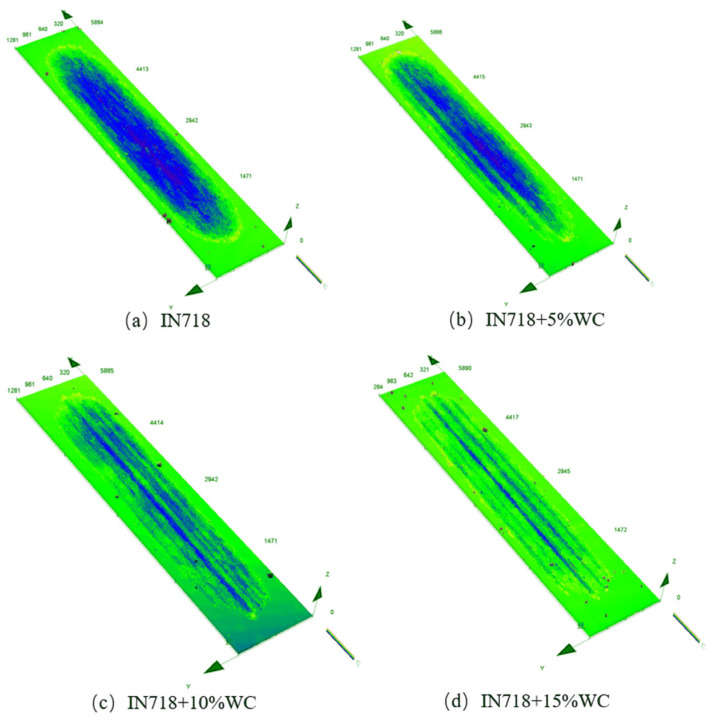
Three-dimensional shape of wear pattern.

Observing the cross-sectional morphology after friction wear using the Olympus OLS4100 revealed the morphology shown in Figure 11. To further investigate the microstructure of WC materials with different WC contents after friction and wear, the wear surface map was converted into a wear depth map, enabling a detailed observation of post-wear microstructural features. Height characterization is shown in Figure 12.

For the pure IN718 coating (Figure 11a), the wear surface remained relatively flat but exhibited characteristics of plastic deformation. This indicates that adhesive wear likely played a role during the initial wear stage, where micro-protrusions on the friction pair surfaces formed adhesive points that were sheared off during relative sliding. However, the significantly higher wear volume compared to the substrate suggests that this adhesive mechanism was quickly superseded by more severe abrasive wear. At 10% WC content, the hard particles provided excellent wear resistance while the matrix retained sufficient toughness to resist crack propagation, resulting in the lowest average coefficient of friction and superior wear resistance. When WC content increased to 15%, excessive hard phases heightened material brittleness, accelerating WC particle shedding. The detached particles transformed into abrasive grains, intensifying micro-plowing. This led to a rebound in the coefficient of friction and a coarser wear surface.

As shown in Figure 11b–d, compared to the relatively uniform wear surface of the pure IN718 coating (Figure 11a), all coatings containing WC particles exhibited distinct grooves on their wear surfaces. These grooves, parallel to the sliding direction, are characteristic features of abrasive wear, specifically resulting from micro-plowing. The formation of these grooves was attributed to the plowing action of the counterface material (Al_2_O_3_ balls), acting as hard abrasive particles on the softer IN718 substrate during friction. More significantly, hard WC particles that have detached or become exposed within the coating function as third-body abrasives. These particles roll and slide between the friction pairs, causing severe plowing effects on both the coating surface and its counterface material. The irregular undulations observed in the three-dimensional topography of Figure 12b–d further confirm the presence of this plowing effect.

**Figure 13:**
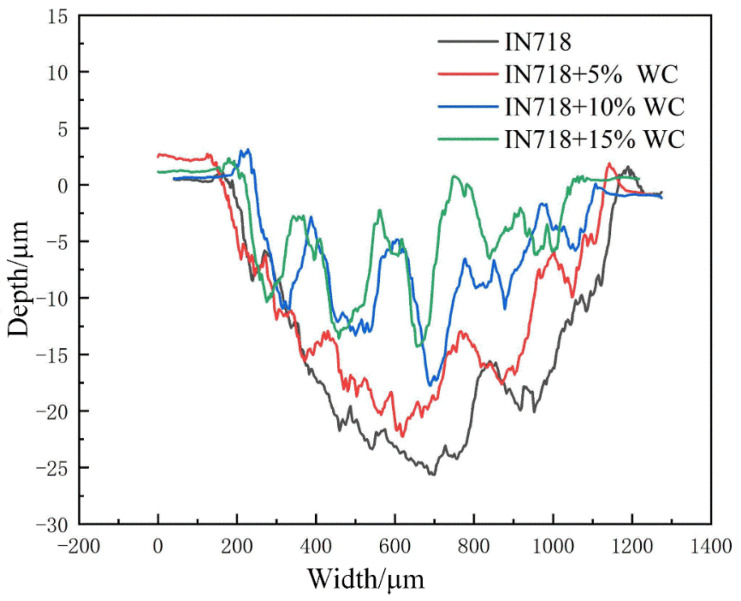
Cross-sectional profile of each specimen after abrasion.

To further characterize the post-wear morphology, cross-sectional images of the worn surfaces were selected and are shown in Figure 13. Among all materials, the substrate material EA4T steel exhibited the highest wear resistance, significantly outperforming IN718 steel in particular. As the WC content increased, the substrate’s wear resistance also gradually improved, reaching its peak at the highest WC content of 15%. Additionally, the wear width of the clad layer remained relatively stable around 900 μm despite increasing WC content. The clad layer depth exhibited a decreasing trend with rising WC content, diminishing from a maximum of 25 μm to 15 μm. As shown in Figure 11c,d, particularly at high WC contents (10% and 15%), microcracking in the matrix material surrounding WC particles and pits left by particle detachment were observable. The mechanism involves microcracks forming at the interface between WC particles and the IN718 matrix under cyclic contact stresses due to mismatched plastic deformation. These cracks propagate and connect, ultimately leading to complete particle detachment or flaking of the matrix material in thin sheets. The increasingly irregular wear cross-section profiles observed in Figure 13 with increasing WC content resulted from the combined effects of this flaking wear and particle detachment.

### 3.4. Tensile Test Results and Fracture Surface Morphology

(1) Tensile Test Results

**Figure 14:**
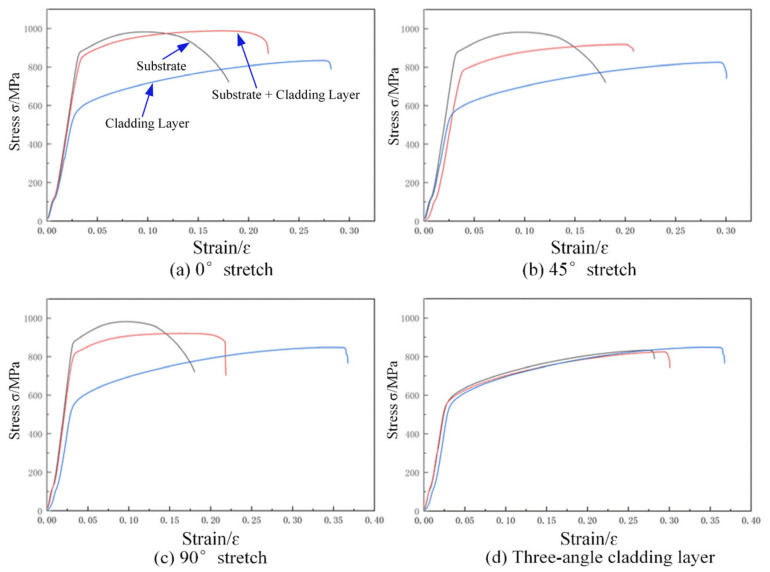
Stress–strain curve at different tension angles.

The stress–strain curves at 0°, 45°, and 90° are shown in Figure 14. All stress–strain curves exhibited continuous and smooth behavior, indicating satisfactory cladding performance under the specified process parameters. No significant defects in the cladding layer or incomplete stress–strain curves resulting from suboptimal process parameters were observed. Preliminary analysis of stress–strain curves at different tensile angles indicates that the tensile strength of the substrate material is significantly higher than that of the cladding layer in various orientations. However, the cladding layer exhibited greater ductility compared to the substrate material.

When subjected to tensile forces in different directions, due to the inherent properties of the materials, the tensile strength sequence is: substrate material > substrate + cladding layer > cladding layer alone. This indicates that the tensile strength of EA4T steel outperforms that of IN718. Specifically, the average tensile strength of the substrate was 977.31 MPa, while the average tensile strength of the cladding layer was 837.03 MPa. Since the substrate and cladding materials each accounted for nearly 50% of the composite structure, the average tensile strength of the substrate-cladding composite lies between those of the substrate and cladding materials. The substrate plays a crucial role in enhancing the tensile strength of the entire composite tensile specimen. At 0°, the tensile strength of the composite component comprising the substrate and the cladding layer outperformed that of the substrate material alone. The average tensile strength of the substrate material was 977.31 MPa, while that of the substrate + cladding layer material averaged 991.49 MPa. Moreover, the range of tensile strength for both materials did not exceed 15 MPa and 25 MPa, respectively. Simultaneously, the substrate and cladding layer exhibited high elongation under elevated stress at 0°. Therefore, for applications with thin cladding layers, aligning the scanning speed direction with the tensile direction can enhance tensile strength. However, for thicker cladding layers, the tensile strength of the 0° cladding layer showed no significant difference from that in other tensile directions.

**Figure 15:**
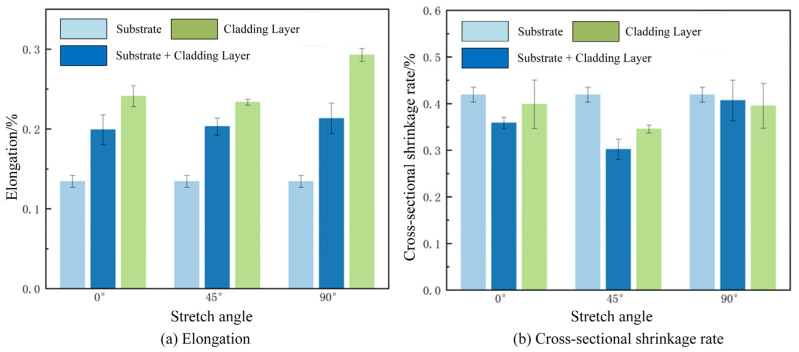
Elongation and section shrinkage after tensile fracture.

Elongation refers to the degree to which a metal can be stretched during tensile testing. A high elongation value indicates that the metal possesses good ductility, meaning it can undergo significant deformation under stress without fracturing. Elongation results are shown in Figure 15a. The substrate + cladding layer composite and pure cladding layer exhibited different elongation values in various directions. Overall, the substrate showed significantly lower elongation than the cladding layer material. The elongation of the substrate + cladding layer composite was intermediate due to the combined effects of both the substrate and cladding layer materials. As the angle varied from 0° to 90°, the elongation of the substrate + cladding layer material was 0.18, 0.20, and 0.19, respectively. Given the isotropic properties of the substrate material, it is considered that the substrate material has a greater influence on the elongation of the substrate + cladding layer material, while the cladding layer at different angles plays a lesser role at this point. Since the pure cladding layer is not influenced by the substrate material, its elongation increases with angle, rising from 0.23 at 0° to 0.29 at 90°. This demonstrates that the loading angle significantly affects elongation, with the tensile direction perpendicular to the scanning speed direction being more conducive to material ductility.

The cross-sectional reduction rate refers to the degree of contraction of the fracture surface relative to the original cross-section after metal fracture during tensile testing. The cross-sectional reduction rate results are shown in Figure 15b. It can be observed that the cross-sectional reduction rate of the substrate was higher than that of the cladding layer material, indicating that the substrate material exhibited a greater degree of necking during fracture. It should be noted that the pure substrate material and pure cladding layer material exhibited different characteristics during tensile testing.

**Figure 16:**
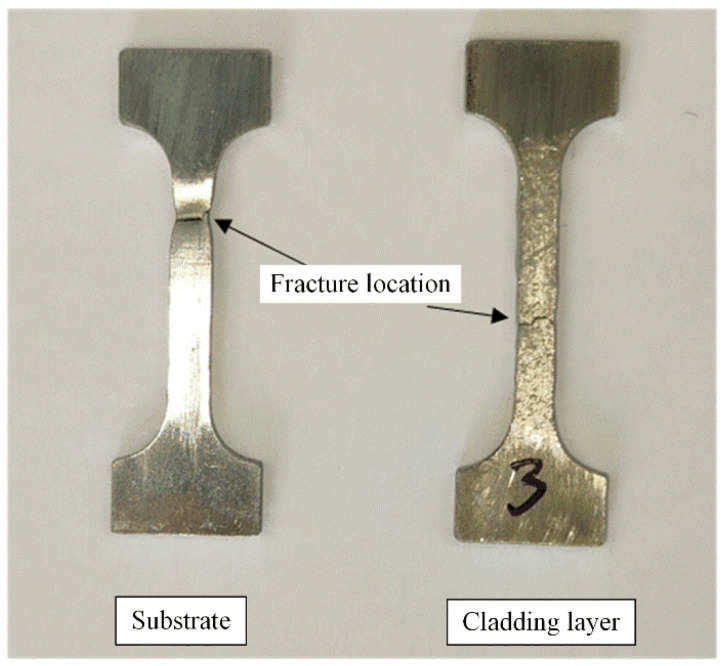
Substrate and cladding specimens after stretching.

Figure 16 displays the post-tensile state of the substrate and 0° cladding specimens.

Distinct characteristics were exhibited by the substrate material and cladding layer material during tensile testing. During tensile loading, the substrate material demonstrated pronounced necking at the fracture location, while the remaining gauge section showed no significant deformation. In contrast, the cladding layer material exhibited substantial plastic deformation across the entire gauge section, resulting in uniformly distributed wrinkles. However, no significant necking occurred at the fracture location. This ultimately resulted in a larger cross-sectional reduction for the substrate material compared to the cladding layer, but a smaller elongation than that of the cladding layer.

**Figure 17:**
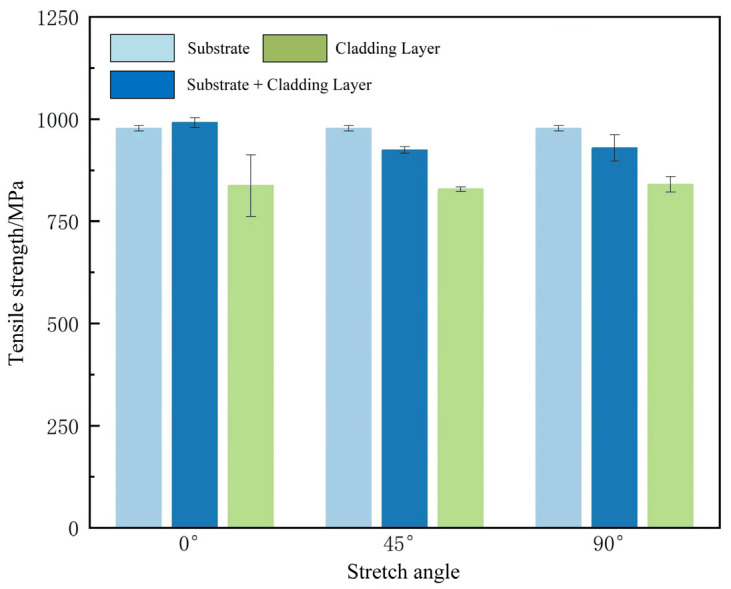
Tensile strength of fused cladding in different tensile directions.

To investigate the properties of the pure cladding layer, the stress–strain curves of the pure cladding layer at three different tensile angles were compared, as shown in Figure 17. The average tensile strengths of the cladding layer at different angles were 977.31 MPa, 828.69 MPa, and 840.44 MPa, respectively. It can be seen that under the experimental parameters employed, the different angles did not affect the tensile properties of the cladding layer.

(2) Analysis of Tensile Fracture Surface Morphology

**Figure 18:**
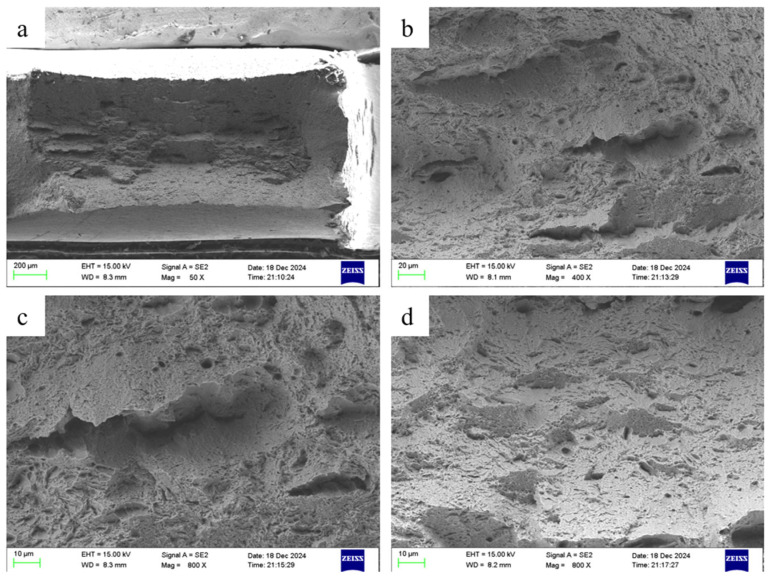
Tensile fracture morphology of substrate material: (**a**) 50×, (**b**) 400×, (**c**) 800×, (**d**) 800×.

Since different materials exhibit distinct properties during tensile testing, fracture mechanisms were determined by analyzing the fracture surfaces of the substrate, substrate + cladding layer, and clad layer after tensile testing. First, the fracture surface of the substrate after tensile testing was analyzed. SEM images are shown in Figure 18.

At 50× magnification, the overall fracture morphology was clearly observable. Necking occurs when localized stress concentration causes significant plastic flow at this point after the material reaches its ultimate tensile strength, ultimately resulting in a reduced cross-sectional area. This indicates that the base material possessed sufficient ductility and toughness. The central region, after undergoing substantial strain, experienced localized stress concentration and deformation. This resulted in microscopic plastic flow within the material, creating a wrinkled appearance overall. At 400× magnification, the aforementioned wrinkles in the central area were identified as cracks originating from plastic fracture. The crack propagation path is related to surrounding defects and grain boundaries in the material, likely caused by stress concentration due to internal inclusions or pores.

Cross-sectional morphology revealed distinct failure patterns between the substrate and cladding layer in the composite material upon tensile fracture. The matrix material exhibited a smoother microstructure compared to the cladding layer, which featured a rough surface with numerous unevenly distributed pores. These pores significantly reduce the tensile strength of the clad layer, as their non-uniform distribution affects various properties. Figure 18a shows that pores not located within the same cross-section are highly likely to serve as crack initiation sites. When selecting and cutting the tensile specimens, the thickness ratio between the substrate and clad layer was essentially maintained at 50% each. However, post-fracture examination revealed the cladding layer occupying a larger cross-sectional area. This discrepancy arises from the differing deformation characteristics of EA4T steel and IN718 during tensile loading. The differing material properties caused greater contraction of the substrate material at the fracture surface. In contrast, the clad layer material underwent uniform deformation throughout the gauge section before fracturing at a specific point within it. However, the fracture location exhibited no significant deformation relative to the entire gauge section. Consequently, this resulted in an uneven distribution of cross-sectional area between the two materials.

**Figure 19:**
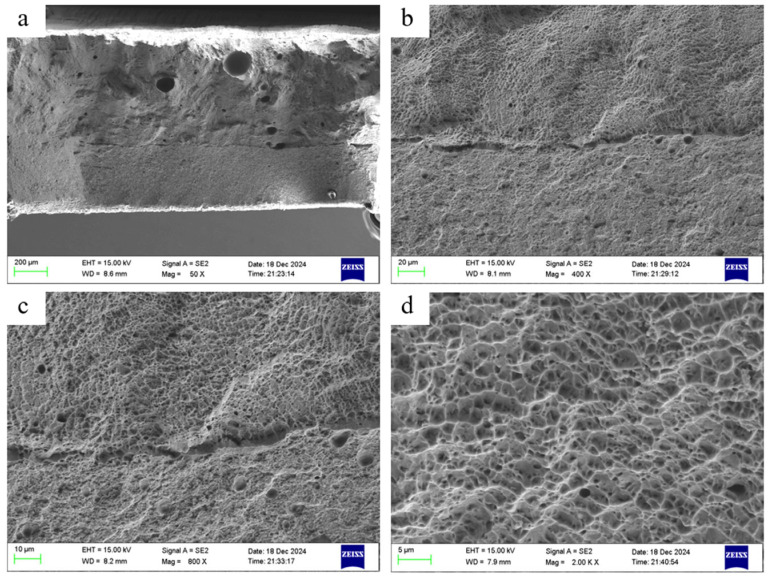
Tensile fracture morphology of substrate and fused cladding bonded parts: (**a**) 50×, (**b**) 400×, (**c**) 500×, (**d**) 2000×.

To investigate the fracture mechanism of the substrate-cladding composite specimen under tensile loading, we analyzed the fracture morphology depicted in Figure 19. Figure 19a presents a low-magnification panoramic view of the fracture surface, clearly revealing two distinct regions: the relatively flat EA4T steel substrate zone on the left and the rough IN718 cladding layer zone on the right. This macroscopic morphology difference directly reflects their distinct mechanical behaviors. The substrate underwent significant plastic deformation and necking prior to fracture, whereas the clad layer exhibited more dispersed overall plastic deformation. At higher magnifications (Figure 19b,c), the microfracture mechanisms of the two materials became more pronounced. Within the clad layer region, extensive dimpling was observed (as shown in Figure 19d). These pits, varying in size and depth, are characteristic markers of micro-pore-aggregated ductile fracture. Their formation mechanism involves micro-pores nucleating at second-phase particles or micro-voids under tensile stress. As plastic deformation intensifies, these micro-pores grow and interconnect, ultimately leading to material separation. This finding fully aligns with the macroscopic mechanical behavior of the IN718 cladding layer exhibiting high elongation, confirming that its failure is predominantly ductile fracture.

In the substrate region, the fracture surface was relatively flatter, but tear edges and shallow dimples were still observable. This indicates that EA4T steel also undergoes ductile fracture, albeit with lower plastic deformation capacity than the clad layer material. In the interface region (right side of Figure 19b), traces of columnar crystals are visible. The columnar grain boundaries formed during laser cladding constitute microstructural weak points. Under external loading, cracks readily propagate along these boundaries, triggering intergranular fracture. This hybrid fracture mode (ductile fracture + intergranular fracture) weakens the load-bearing capacity of the interface region, constituting a key factor influencing the performance of the composite specimen.

The microstructures of the cladding layer and the substrate material exhibited distinct differences. The cladding layer material displayed a granular structure due to the laser cladding process. Even in Figure 19b, columnar grains distributed along the cross-section can be observed at the right boundary between the cladding layer and the substrate. Additionally, the laser cladding process caused grains to grow at random angles within the melt pool, resulting in microstructural inhomogeneity within the cladding layer and subsequent uneven stress distribution. Consequently, higher magnification revealed that the cladding layer exhibited a rougher surface texture than the substrate. At the interface between substrate and cladding layer, asynchronous deformation during tensile loading induced dislocations, forming a dislocation crack that traversed both the substrate and cladding layer. At higher magnifications, the clad layer exhibited numerous irregularly shaped pits of varying sizes and depths, interlaced without distinct dendritic patterns—characteristic features of ductile pits. These pits formed through plastic deformation during tensile stress, coupled with the nucleation and growth of micro-voids.

**Figure 20:**
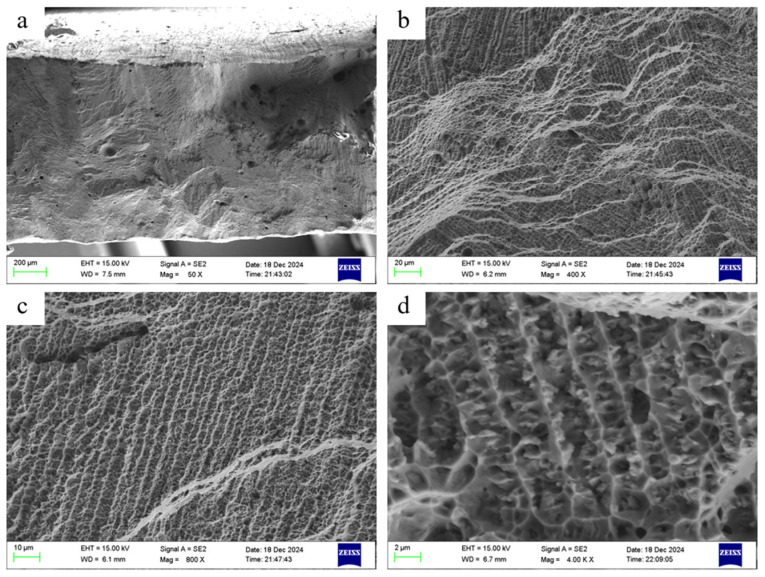
Tensile fracture morphology of fused cladding layer: (**a**) 50×, (**b**) 400×, (**c**) 800×, (**d**) 4000×.

The cladding layer exhibited surface irregularities after tensile testing. The cross-sectional state after 0° tensile testing is shown in Figure 20. Numerous defects were observable within the fracture surface. These defects were not uniformly distributed across the same cross-section; rather, their presence led to reduced tensile stress capacity in the surrounding areas, ultimately manifesting as fractures. Figure 20b reveals a distinct textured pattern in the fracture surface, with grain orientations ranging from 45° to 135°. Additionally, certain banded structures resembling columnar crystal morphology were clearly visible at specific locations. To investigate whether these structures represent columnar crystals or are related to them, ImageJ software revealed an average width of 3.17 μm—consistent with the columnar crystal dimensions described earlier. This confirms that the structures depicted are columnar crystal formations. The growth direction of the columnar crystal structure shown in Figure 20b ranges from 45° to 135°, suggesting that columnar crystal growth during laser cladding is concentrated within this range. A more detailed examination of the columnar crystal structure after tensile fracture yielded Figure 20d. This reveals that “voids” were created when the columnar crystals were pulled out during specimen fracture, indicating complete “extraction” of the crystals. The rapid growth of columnar crystals during laser cladding resulted in relatively weak intergranular bonding. Under external stress, stress concentration occurs at grain boundaries, causing them to fracture first and initiating intergranular fracture.

## 4. Conclusions

This study systematically investigated the mechanical properties of IN718 and IN718-WC composite coatings fabricated by laser cladding on EA4T steel. The key findings, fully supported by the experimental data, are summarized as follows:

1. The addition of WC particles effectively addressed the insufficient hardness of the IN718 coating. The microhardness increased significantly with WC content, reaching approximately 130% of the pure IN718 value at 15 wt% WC. The hardening mechanism was attributed to both solid solution strengthening and the presence of unmelted WC particles.
2. The friction and wear tests demonstrated that the wear volume decreased progressively with increasing WC content. However, the average coefficient of friction reached a minimum at 10 wt% WC. This optimal performance is attributed to a balanced combination of effective reinforcement and minimal stress concentration, as evidenced by the surface and cross-sectional wear morphology analysis.
3. Tensile tests at different angles revealed clear anisotropic behavior. The substrate material exhibited superior strength but lower ductility compared to the cladding layer. For the pure IN718 cladding layer, while tensile strength showed minimal variation with angle, the elongation was significantly highest at 90°.
4. Detailed microstructural investigations into the stability, solubility, and distribution of WC reinforcing phases within the IN718 matrix, utilizing advanced characterization techniques, constitute a critical and necessary focus of our ongoing and future studies to fully elucidate the underlying strengthening and wear-resistant mechanisms.

In summary, the experimental data presented in this work provide a solid foundation for selecting WC content and scanning strategy to tailor the mechanical performance of laser-cladding IN718 coatings for specific service requirements.

## Figures and Tables

**Table 1:** Mass Fraction of Chemical Composition for EA4T Steel (wt%) [21].

C	Si	Mn	P	S	Cr	Ni	Cu	Mo	V	Fe
0.25	0.27	0.68	0.017	0.004	1.09	0.08	0.05	0.23	0.002	allowance

**Table 2:** Mass Fraction of Chemical Composition for IN 718 (wt%) [21].

C	Cr	Ni	Co	Mo	Al	Nb	Fe
0.08	20.00	52.00	1.00	3.00	0.5	5.00	allowance

**Table 3:** WDW-100E electronic universal testing machine technical parameters.

Technical Indicators	Parameters
Maximum load	100 kN
Maximum stretch space	500 mm
Test speed range	0.01–350 mm/min
Test Force Accuracy	±0.5%
Displacement resolution	0.01 mm

**Table 4:** Differences in elemental content at different locations.

No.	Ni (wt%)	Cr (wt%)	Fe (wt%)	Mo (wt%)	Ti (wt%)	Nb (wt%)
Point 1	52.33	19.51	21.53	3.01	0.61	2.67
Point 2	49.08	16.01	15.60	4.08	1.43	13.55
Point 3	52.71	19.41	21.16	2.90	0.79	2.71
Point 4	51.26	17.68	17.10	3.54	1.45	8.66
Point 5	53.08	19.18	21.51	2.34	0.71	2.80

## Data Availability

The original contributions presented in this study are included in the article. Further inquiries can be directed to the corresponding author.
